# Differences in lumbar spine and lower extremity kinematics in people with and without low back pain during a step-up task: a cross-sectional study

**DOI:** 10.1186/s12891-017-1721-z

**Published:** 2017-08-25

**Authors:** Katie Mitchell, Madeline Porter, Lauren Anderson, Carter Phillips, Grayson Arceo, Brian Montz, Susan Levy, Sara P. Gombatto

**Affiliations:** 1 San Diego State University Doctor of Physical Therapy Program, San Diego, CA USA; 2 San Diego State University School of Exercise and Nutritional Sciences, 5500 Campanile Drive, San Diego, CA 92182-7251 USA

**Keywords:** Kinematics, Low back pain, Lumbar spine, Lower extremities, Functional

## Abstract

**Background:**

Low back pain (LBP) affects more than one third of the population at any given time, and chronic LBP is responsible for increased medical costs, functional limitations and decreased quality of life. A clear etiology is often difficult to identify, but aberrant posture and movement are considered contributing factors to chronic LBP that are addressed during physiotherapy intervention. Information about aberrant movement during functional activities in people with LBP can help inform more effective interventions. The purpose of this study was to determine if there are differences in lumbar spine and lower extremity kinematics in people with and without LBP during a step-up task.

**Methods:**

A convenience sample of 37 participants included 19 with LBP and 18 without a history of LBP. All participants were between the ages of 18 and 65, and controls were matched to participants with LBP based on age, gender and BMI. A motion capture system was used to record spine and lower extremity kinematics during the step-up task. ANOVA tests were used to determine differences in three-dimensional kinematics between groups.

**Results:**

Participants with LBP displayed less lower lumbar motion in the sagittal plane (*P* = 0.001), more knee motion in the coronal plane (*P* = 0.001), and more lower extremity motion in the axial plane (*P* = 0.002) than controls.

**Conclusions:**

People with LBP display less lower lumbar spine motion in the sagittal plane and more out-of-plane lower extremity motion. Clinically, the step-up task can be used to identify these aberrant movements to develop more focused functional interventions for patients with LBP.

**Trial registration:**

Not applicable.

## Background

Low back pain (LBP) is a chronic epidemic affecting up to 36% of the population in any given year [1], and up to 80% of people at some point during their lives [2]. In a systematic review on the global burden of disease, LBP ranked highest in disability and sixth in overall burden [1]. In the United States, the estimated annual cost of LBP exceeds $100 billion [3]. In addition to being costly, LBP affects quality of life by resulting in missed work, decreased activity, and increased difficulty in caring for children [1]. It is often difficult to identify the source of LBP, and cases that have no abnormality detected with radiology are typically classified as nonspecific LBP [4, 5]. However, investigators and clinicians have proposed that movement abnormalities may contribute to the development and recurrence of LBP [6, 7]. Identifying abnormal movements in people with LBP may lead to more focused and effective movement-based interventions.

A substantial amount of research has been conducted to identify movement impairments in people with LBP during simple trunk and limb movements [8–14]. This information is useful when conducting and interpreting the findings from a clinical examination. Some of these clinical tests include forward bending, trunk lateral bending, prone extension, prone hip extension, knee flexion, and hip lateral rotation [8–14]. A limited number of studies report on lumbar spine kinematics during functional tasks, which may be more representative of everyday activities [13, 15–18]. Investigators have evaluated functional tasks such as stair climbing, putting on a sock, lifting and transferring a box, and picking objects up from the floor [13, 15–18]. Although measurements of interest and findings vary across these studies of functional tasks, investigators have generally reported that people with LBP display less sagittal and lateral flexion, but greater amounts of axial rotation when compared to people without LBP.

Despite the existing evidence on lumbar spine kinematics during clinical tests and functional tasks, there are several important factors that have not been consistently considered. First, much of the previous research has considered the lumbar spine a single rigid segment [13, 15, 16, 19–21]. Measuring the spine as a rigid segment can be problematic, as it does not account for independence of movement within different regions of the lumbar spine. Further, Mitchell et al. reported that the upper and lower lumbar regions display differential movement characteristics [17]. Last, few investigators have characterized lower extremity movement during functional tasks in people with LBP. Crosbie et al. and Shum et al. have reported that people with a history of LBP displayed altered lumbar-hip coordination compared to the control group [16, 22, 23]. Based on these findings, more research is needed to examine both lumbar spine and lower extremity kinematics during functional tasks.

The purpose of this study was to determine if there are differences in regional lumbar spine and lower extremity kinematics in people with and without LBP during a step-up task. We hypothesized that people with LBP would demonstrate less overall lumbar flexion, but greater lower lumbar flexion and axial rotation during this task. Additionally, we hypothesized that there would be differences in lower extremity kinematics between people with and without LBP.

## Methods

A cross-sectional observational design was used to determine differences in kinematics between people with and without LBP.

### Subject characteristics

A power analysis was conducted based on data from a pilot study of group differences in kinematics during a walking and a pickup task (partial n^2^ = 0.338) [24, 25]. We determined that a minimum of 15 subjects was needed to detect a group by region interaction effect with 80% power. For the current study, 37 subjects between the ages of 18 and 65 participated. Nineteen subjects had LBP, and 18 subjects had no history of LBP (controls). The subjects with LBP were recruited from 2 outpatient physical therapy clinics, where they were seeking treatment for their LBP. The controls were recruited from physicians’ offices and the surrounding community. Subjects with LBP reported a primary complaint of LBP that limited activities of daily living for at least 3 consecutive days. Exclusion criteria included a history of serious spinal or other medical conditions, physical or mental disabilities, pregnancy, persons who are unable to provide their own legal informed consent, and a BMI > 30 kg/m^2^. The body mass index criterion was selected because subcutaneous tissue may influence the accuracy of skin marker placement and tracking. Subject recruitment and testing occurred from January 2011 – May 2013. The Human Subjects Research Committee at Nazareth College in Rochester, NY, approved the protocol and the subjects provided informed consent before participating.

To measure the level of pain, disability, and fear avoidance, each subject with LBP completed a Numeric Rating Scale for Pain (NRS), LBP symptom questionnaire, Oswestry Disability Index (ODI), and Fear-Avoidance Beliefs Questionnaire (FABQ) prior to participation [26–29].

### Motion capture

**Fig. 1:**
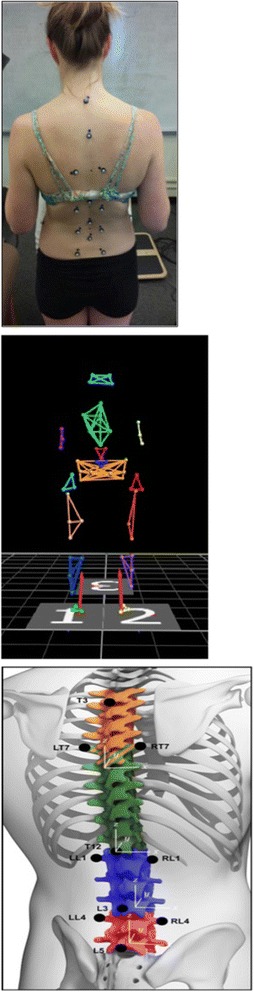
Lumbar spine marker set and model. a. Marker Placement on a participant. b. Computer Model as shown in the data collection software program. c. Spine Model representation with upper (blue) and lower (red) lumbar segments identified**Reprinted from *Gait & Posture*, 42(4); Gombatto SP, Brock T, DeLork A, Jones G, Madden E, Rinere C, *Lumbar spine kinematics during walking in people with and people without low back pain;* 539–544, 2015, with permission from Elsevier (https://www.journals.elsevier.com/gait-and-posture/) [30]

A 9-camera, 3-dimensional optical motion capture system (Vicon Inc., Denver, CO) was used to capture data using a frame rate of 100 Hz as subjects performed the step-up task. Fifty-seven retro-reflective markers were placed on pre-determined anatomical landmarks of the lumbar spine, pelvis and extremities (Fig. 1). The landmarks were located, and then markers were placed on all subjects by the same licensed physical therapist, who had 10 years experience with motion capture testing. The lumbar spine model has been described in detail in previous publications, and was tested for and demonstrated acceptable reliability and validity for measuring regional lumbar spine kinematics [10, 25, 30]. The upper lumbar segment was defined by markers 4 cm lateral to L1 and on the spinous process of L3. The lower lumbar segment was defined by markers 4 cm lateral to L4 and on the spinous process of L5. Markers on the PSIS, ASIS, and iliac crest bilaterally defined the pelvis. For the thigh segment, markers were placed on the superior, inferior and posterior thigh. For the lower leg segment, markers were placed on the fibular head, superior, inferior and posterior lower leg and the tibial tuberosity. Markers on the feet were placed on the third metatarsal head, fifth metatarsal tuberosity, and calcaneus. Markers were placed bilaterally on the medial and lateral femoral condyles and medial and lateral malleoli for calibration, but were removed for the movement trials.

### Movement testing

Prior to conducting each step-up trial, a static calibration trial was captured to calculate knee and ankle joint centers and a dynamic trial was captured to calculate functional hip joint centers. For the step-up trial, each subject was instructed to step-up onto a single rectangular step, 24 cm tall, placed ~13 cm from the great toe (Fig. 1). A higher than normal (15–20 cm) step height was used to challenge the subject and to accentuate any movement impairments. The subject was instructed to step-up with the right foot, followed by the left, and then remain on the step. The test movement was repeated three times leading with the right foot, and then three times leading with the left foot. Subjects were allowed to move at a self-selected speed, with no additional instructions. Each subject rated their LBP on the 11-point NRS (0 = no pain, 10 = worst imaginable pain) before and after performing the step-up task; change in pain behavior on the NRS was recorded.

### Data processing

The marker data was post-processed, without knowledge of group membership, using Nexus software (Vicon, Inc., Denver, CO) to correct unlabeled trajectories and fill gaps using the gap filling function in Nexus. Data was exported from Nexus and then imported to Visual 3D (C-Motion, Inc., Germantown, MD) where marker data were filtered using a second-order Woltring filter (GCVSPL) [31] (2 × 10^−4^ MSE variance; optimization mode, 3) and the custom spine model was applied [10, 32]. Based on the parameters used for the GCVSPL filter, and the 100 Hz sampling frequency, the equivalent cutoff frequency parameter for a 2nd order low-pass Butterworth filter would be approximately 4.2 Hz (3.4 Hz with adjustment for dual pass) [33]. https://isbweb.org/software/sigproc.html] Functional joint centers were calculated for the hip from the dynamic trial and anatomic joint centers were calculated for the knee and ankle from the static calibration trial [34]. The upper lumbar, lower lumbar, hip, knee and ankle segment angles were derived in the sagittal, coronal, and axial planes from the start to end of the movement task. The start of the task was identified when the movement of the T1 marker exceeded 5% of its maximum linear velocity in any direction. The end of the task was identified when the calcaneus marker on the trailing limb reached a linear velocity of zero in the vertical direction, and both feet were on the step. Movement time was calculated and maximum and minimum segment angles were identified between the start and end of the movement task. Angle excursion for each segment was calculated as the difference between the maximum and minimum segment angle in each of the three planes of motion for each trial. Angle excursion measures from each trial then were averaged across the three right step-up trials and then across the three left step-up trials.

### Data analysis

The data were analyzed using SPSS version 21.0 using an alpha level of 0.05. Group differences in subject characteristics and movement time were evaluated using t-tests for ratio scale data and chi-square tests for categorical data.

All lumbar spine and lower extremity kinematic measures were examined for normality and the presence of outliers. For each measure, standardized skewness and standardized kurtosis were calculated to assess normality, as these would best highlight departures from normality beyond what is tolerated in the generally robust analysis of variance (ANOVA) [35]. Kinematic measures with standardized skewness or kurtosis values outside the accepted range for normality (±2) were transformed using the log natural transformation (base-e logarithm) for analysis to ensure that any non-normality issues did not threaten statistical findings.

Separate group X region mixed design ANOVAs were conducted for the lumbar spine (2 groups × 2 regions) and lower extremities (2 groups × 3 regions) and for each plane of motion (sagittal, coronal, axial). Group (LBP, control) served as a between subject factor and region (*lumbar spine*: upper, lower; *lower extremity*: hip, knee, ankle) served as the repeated measures factor. Preliminary analysis including a repeated measures factor of side revealed no effect of step-up side, so measures were averaged for the right and left step-up tasks in subsequent analyses. Because group differences were of primary interest in the current study, significant interactions were followed up with simple nested comparisons comparing the two groups to further examine the source of the significant interactions. Data were back transformed to report in graphical form and to report confidence intervals.

## Results

There were no group differences in subject characteristics or movement time (*P* > 0.05, Table 1).

**Table 1:** Subject Demographics and Movement Time, Mean (SD)

	LBP (*N* = 19)	Control (*N* = 18)
*Age*	30.0 (15.3)	26.1 (8.6)
*Gender*	12 Females, 7 Males	10 Females, 8 Males
*Height (cm)*	169.7 (11.2)	167.8 (12.5)
*Weight (kg)*	71.6 (15.0)	72.0 (14.5)
*BMI (kg/m* ^*2*^ *)*	24.4 (2.9)	25.5 (3.6)
*Step-Up Movement Time (seconds)*	3.6 (0.4)	3.5 (0.5)

Characteristics of the low back pain group are presented in Table 2.

**Table 2:** Characteristics of the low back pain group (*N* = 19)

	Mean (Standard Deviation)	Median	Range
*Episodes of pain in last 12 months*	3.7 (4.1)	3.5	14.0
*Visual analog scale pain scale (0-100 mm)*	27.4 (21.9)	42.5	59.0
*Average pain for last 7 days (0–10)*	3.7 (2.4)	5.0	6.0
*Current Pain (0–10)*	2.1 (1.8)	3.0	4.0
*Modified Oswestry (0–100%)*	17.7 (12.4)	22.0	24.0
*FABQ-PA (0–24)*	13.4 (4.4)	13.5	10.0
*FABQ-W (0–42)*	11.3 (8.1)	10.5	10.0

*FABQ-PA* Fear Avoidance Belief Questionnaire – Physical Activity Scale

*FABQ-W* Fear Avoidance Belief Questionnaire – Work Scale

### Kinematic measures

Standardized skewness and kurtosis values indicated departures from normality for skewness for coronal plane excursion of the knee (standardized skewness = 2.75), sagittal plane (standardized skewness = 2.20) and axial plane (standardized skewness = 3.13) excursion of the lower lumbar region. ANOVA models are generally robust to violations of normality, however to ensure the reliability of test-statistics, data were log transformed prior to conducting analyses [35]. This resolved non-normality as reflected by standardized skewness and kurtosis values for all measures falling within ±2.

### Lumbar spine kinematics

**Fig. 2:**
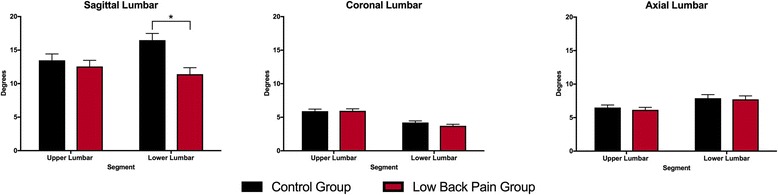
Mean (SE) differences in 3D kinematics of the upper and lower lumbar region, during a step-up functional task in subjects with and without low back pain. * indicates a statistically significant effect of group from post-hoc tests (*P* = .001)

Results for group differences in lumbar spine kinematics are presented in Fig. 2 and results of statistical tests are presented in Table 3. In the sagittal plane, there was a significant group by region interaction (*P* = 0.016) and a significant main effect of group (*P* = 0.027). There was no significant main effect of region (*P* = 0.287). Follow up nested comparisons revealed that subjects in the LBP group displayed less sagittal plane movement in the *lower* lumbar spine than the control group (mean diff: −5.1°; 95% CI: -7.9° − −2.2°; *P* = 0.001), but there was no group difference in *upper* lumbar spine motion in the sagittal plane (mean diff: −0.9°; 95% CI: -3.6° − 1.8°; *P* = 0.603). In the *coronal* and *axial* planes respectively, there were no significant main effects of group (*P* = 0.418, *P* = 0.555) or group by region interactions (*P* = 0.169, *P* = 0.767).

**Table 3:** Results of overall and post-hoc ANOVA tests for lumbar spine and lower extremity kinematics

			Overall	Post-Hoc^a^		Overall	Post-Hoc^a^		
Plane of Motion	Effects	Statistical Values	Lumbar Spine	Upper Lumbar	Lower Lumbar	Lower Extremity	Hip	Knee	Ankle
Sagittal	Region	F-statistic	1.167			2039.326			
		*P*-value	0.287			*0.000			
		eta^2^	0.032			0.983			
	Group	F-statistic	5.354	0.275	12.385	0.003	1.783	2.01	2.825
		*P*-value	*0.027	0.603	0.001*	0.957	0.190	0.165	0.102
		eta^2^	0.133	0.008	0.261	0.001	0.048	0.054	0.075
	Group x Region	F-Statistic	6.400			5.190			
		*P*-value	*0.016			*0.008			
		eta^2^	0.155			0.129			
Coronal	Region	F-statistic	79.118			56.301			
		*P*-value	*0.001			*0.000			
		eta^2^	0.693			0.617			
	Group	F-statistic	0.673			15.207	0.378	12.619	1.908
		*P*-value	0.418			*0.000	0.543	*0.001	0.176
		eta^2^	0.019			0.303	0.011	0.265	0.052
	Group x Region	F-Statistic	1.972			6.022			
		*P*-value	0.169			*0.004			
		eta^2^	0.053			0.147			
Axial	Region	F-statistic	18.098			4.4145			
		*P*-value	*0.001			*0.020			
		eta^2^	0.341			0.106			
	Group	F-statistic	0.355			10.828			
		*P*-value	0.555			*0.002			
		eta^2^	0.010			0.236			
	Group x Region	F-Statistic	0.089			0.480			
		*P*-value	0.767			0.621			
		eta^2^	0.003			0.014			

^a^Post-hoc tests were simple nested comparison follow-ups to significant interactions

*Indicates statistically significant *p*-value < 0.05

### Lower extremity kinematics

**Fig. 3:**
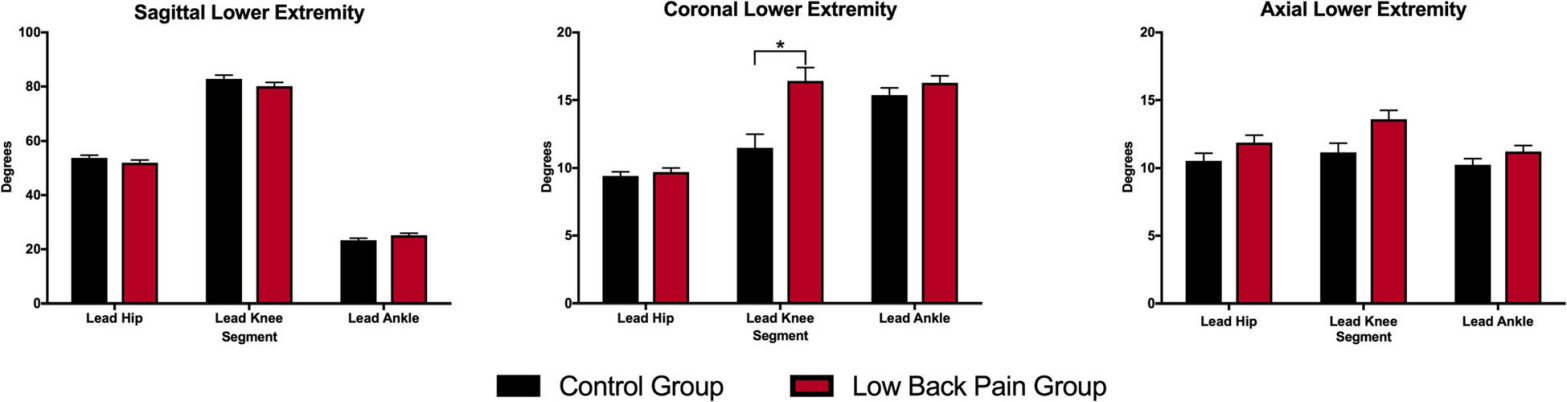
Mean (SE) differences in 3D kinematics of the lower extremity, during a step-up functional task in subjects with and without low back pain. * indicates a statistically significant effect of group from post-hoc tests (*P* = .001)

Results for group differences in lower extremity kinematics are presented in Fig. 3 and results of statistical tests are presented in Table 3. In the *sagittal* plane, there was a significant group by region interaction effect (*P* = 0.008) and a significant main effect for region (*P* = 0.001). There was no significant main effect of group (*P* = 0.957). Follow up nested comparisons revealed no significant group differences in lower extremity kinematics at the hip (mean diff: −1.8°; 95% CI: -4.6° − 1.0°; *P* = 0.190), knee (mean diff: −2.7°; 95% CI: -6.6° − 1.3°; *P* = 0.165), or ankle (mean diff: 1.9°; 95% CI: -0.3° − 4.0°; *P* = 0.102) in the sagittal plane. In the *coronal* plane, there was a significant group by region interaction effect (*P* = 0.004) and a significant main effect of group (*P* = 0.001) and region (*P* = 0.001). Follow up nested comparisons revealed that the LBP group displayed significantly more coronal plane movement at the knee joint than the control group (mean diff = 4.9°, 95% CI: 2.0°-7.8°, *P* = 0.001), but there were no group differences in coronal plane movement at the hip (mean diff: 0.3°; 95% CI: -0.6° − 1.2°; *P* = 0.543) or ankle joint (mean diff: 0.7°; 95% CI: -0.6° − 2.4°; *P* = 0.176). In the *axial* plane, there was a significant main effect of group (*P* = 0.002) and region (*P* = 0.020), but no significant group by region interaction effect (*P* = 0.621). Subjects in the LBP group displayed more axial plane lower extremity movement than controls.

## Discussion

Subjects in the LBP group displayed less sagittal plane movement in the lower lumbar spine and more out-of-plane motion at the lower extremity during the step-up task. Based on the prior literature, we had hypothesized that subjects with LBP would display less sagittal plane movement in the lumbar spine as a whole, but more sagittal plane movement in the lower lumbar spine. However, we found that subjects with LBP displayed less lumbar flexion, specifically in the lower lumbar spine. Less sagittal plane motion in the lumbar spine is consistent with previous studies on kinematics during a step-up task that modeled the lumbar spine as a single rigid segment [15]. Lee et al. examined single-step and double-step stair climbing, and reported that people with LBP demonstrated less flexion and extension in the lumbar spine during the task than the control group [15]. In the current study, less sagittal plane movement was found specifically in the lower lumbar spine.

There are several possible explanations for the group differences. An individual with LBP may attempt to avoid pain with muscle guarding [36]. There may also be changes at the intervertebral junction that result in stiffening and decreased excursion. Last, inadequate activation of spinal flexors may result in decreased movement at the lower lumbar spine during the step-up task. Future studies could examine trunk muscle EMG, or include MRI to determine which factors may contribute to the reported group differences in kinematics. This information may be useful for specifying interventions that target factors contributing to movement impairments.

Also, we had hypothesized that people with LBP would display significantly greater axial rotation in the lumbar spine, but they did not. There are several potential explanations for why our results differed from our hypothesis and from the prior literature. Some of the prior studies included analysis of functional tasks in a seated position, which limits pelvic motion and may minimize lower extremity contribution to the task [16]. In the current study, subjects were standing when performing the step-up task and the lower extremities were free to move. During the step-up task, subjects may have compensated for less lumbar spine motion with lower extremity movement. This is in contrast to the increases in axial rotation at the lumbar spine that was observed in the prior research [16].

It is important to note that some investigators have used a different approach to evaluate kinematics during trunk movements and functional tasks in people with LBP by examining movement variability rather than segment excursion. Movement variability has been proposed as an index of motor control, such that greater variability represents impaired control of movement. However, movement variability has been defined and measured in a number of different ways across studies and may describe either trial-to-trial variability or within trial variability. Further, the effect of LBP on movement variability is conflicting. Some researchers have noted that people with LBP display *greater* movement variability than controls for some movement tasks [13, 14, 37]. Conversely, other investigators have reported that people with LBP display less variability of movement or muscle activation during movement tasks in people with LBP [18, 38, 39]. In a study of kinematic variability during gait, Lamoth et al. reported that movement variability in subjects with LBP during gait depended on the plane of motion that was evaluated; people with LBP displayed less kinematic variability in the transverse plane and greater variability in the coronal plane [40]. Movement variability was not evaluated in the current study, but examining movement variability in the upper and lower lumbar spine and extremities during functional tasks has not been widely examined and could be an area of future investigation to further evaluate movement control during functional tasks.

We also hypothesized that groups would display differences in lower extremity kinematics, and subjects with LBP displayed greater out-of-plane lower extremity motion than controls. Despite extensive research on the strong relationship between hip and lumbar spine movement, to our knowledge, few investigators who have examined lumbar spine movement during functional tasks, have evaluated movement throughout the lower extremity. A possible explanation for differences in coronal and axial plane lower extremity movement between subjects with and without LBP is regional interdependence. Subjects with LBP may be compensating for lack of movement in the lumbar spine, with out-of-plane movement at the lower extremity. In one previous study, investigators reported that subjects with recurrent LBP displayed more hip motion when compared to the control subjects in order to complete a reaching task. These prior findings and the current findings indicate that lower extremity motion can be influenced by spine motion and LBP [23]. When people with LBP use more lower extremity movement to accomplish functional tasks, particularly in the coronal and axial planes, they may be increasing their risk of secondary lower extremity injury. Previous investigators have linked lumbopelvic control problems and LBP with increased risk of lower extremity injuries [41, 42]. Data from the current study may suggest a potential mechanism for this increased risk.

Due to the heterogeneous nature of LBP, and that investigators have reported differences in movement characteristics among movement-based LBP subgroups [9, 43], it would be beneficial to examine differences in lumbar spine kinematics during functional tasks among movement-based LBP subgroups. In this study, we did not have an adequate sample size in the LBP group to statistically evaluate differences among movement-based LBP subgroups. Future researchers and clinicians may need to consider the potential for these movement-based subgroup differences during performance of functional tasks.

Based on the findings of the current study, the step-up task may be used as an assessment tool to identify abnormal movements and focus the physical therapy evaluation and treatment towards affected regions. Based on the data from the current study, interventions for people with LBP could include facilitating mobility at the lumbar spine and controlling movement at the lower extremities, while considering the patient response and tolerance. The difference in kinematics observed between groups in this study was approximately 5 degrees. Given that a physical therapist has extensive training and expertise in movement observation, it is likely that he or she would be able to detect such differences in movement when observing a patient performing functional tasks in a clinical setting. Identifying aberrant movements in patients with LBP during functional activities may lead to a more focused and individualized movement-based intervention.

### Study limitations

There are several limitations to the current study. First, because this is a cross-sectional study, we were unable to determine whether lumbar spine kinematics in the LBP group contributed to the LBP problem or occurred as a result of the LBP problem. However, subjects were recruited from a physical therapy clinical population and therefore the findings can be generalized to patients in this setting. Second, there is the potential for error in measuring spine movement with surface marker measures because of skin movement. Alternatively, surface marker measures are currently the only available method to objectively measure both lumbar spine and lower extremity kinematics during complex functional tasks. Last, some studies have shown that people with LBP have differences in movement variability, but the kinematic variables used in the current study were segment excursions and we did not measure movement variability.

## Conclusions

Subjects with LBP display less movement in the sagittal plane in the lower lumbar spine and more out-of-plane motion in the lower extremities during a step-up task. The greater degree of out-of-plane motion in the lower extremities may be compensation for lack of motion at the lumbar spine in order to accomplish the task. Identifying abnormal movements in people with LBP may help to focus evaluation and treatment of movement-based impairments.

## Abbreviations

FABQ: Fear-Avoidance Beliefs Questionnaire
LBP: Low back pain
NRS: Numeric Rating Scale for Pain
ODI: Oswestry Disability Index
